# Polygenic Risk Score Is Associated with Developing and Dying from Lung Cancer in the National Lung Screening Trial

**DOI:** 10.3390/jcm14093110

**Published:** 2025-04-30

**Authors:** Robert P. Young, Raewyn J Scott, Tom Callender, Fenghai Duan, Paul Billings, Denise R. Aberle, Greg D. Gamble

**Affiliations:** 1 Faculty of Medical and Health Sciences, University of Auckland, Auckland P.O. Box 37-971, New Zealand; rhopkins@adhb.govt.nz (R.J.S.); gd.gamble@auckland.ac.nz (G.D.G.); 2 Respiratory Research Group, Greenlane Clinical Centre, Epsom, Auckland 1344, New Zealand; 3 Department of Applied Health Research, University College London, London WC1E6B1, UK; t.callender@ucl.ac.uk; 4 Department of Biostatistics and Centre for Biostatistics and Health Data Science, Brown University of Public Health, Providence, RI 02912, USA; fduan@stat.brown.edu; 5 Natera Inc., Austin, TX 78753, USA; pbillings@natera.com; 6 Department of Radiological Sciences, David Geffen School of Medicine, UCLA, Los Angeles, CA 90095, USA; daberle@mednet.ucla.edu

**Keywords:** lung cancer, screening, polygenic risk score, outcomes

## Abstract

**Highlights:**

**What are the main findings?**

- In this proof-of-concept study, a lung cancer PRS helped predict who develops and dies of lung cancer (clinical validity).
- This was independent of the patient’s clinical risk variables (smoking, age and comorbidity), lung function, lung cancer characteristics (histology, stage and surgery), screening arm and family history.

**What is the implication of the main findings?**

- In a lung cancer screening trial, a PRS predicted lung cancer lethality/mortality and may represent a novel biomarker of lung cancer outcomes following screening.

**Abstract:**

**Background:** Epidemiological studies suggest lung cancer results from the combined effects of smoking and genetic susceptibility. The clinical application of polygenic risk scores (PRSs), derived from combining the results from multiple germline genetic variants, have not yet been explored in a lung cancer screening cohort. **Methods:** This was a post hoc analysis of 9191 non-Hispanic white subjects from the National Lung Screening Trial (NLST), a sub-study of high-risk smokers randomised to annual computed tomography (CT) or chest X-ray (CXR) and followed for 6.4 years (mean). This study’s primary aim was to examine the relationship between a composite polygenic risk score (PRS) calculated from 12 validated risk genotypes and developing or dying from lung cancer during screening. Validation was undertaken in the UK Biobank of unscreened ever-smokers (N = 167,796) followed for 10 years (median). **Results:** In this prospective study, we found our PRS correlated with lung cancer incidence (*p* < 0.0001) and mortality (*p* = 0.004). In an adjusted multivariable logistic regression analysis, PRS was independently associated with lung cancer death (*p* = 0.0027). Screening participants with intermediate and high PRS scores had a higher lung cancer mortality, relative to those with a low PRS score (rate ratios = 1.73 (95%CI 1.14–2.64, *p* = 0.010) and 1.89 (95%CI 1.28–2.78, *p* = 0.009), respectively). This was despite comparable baseline demographics (including lung function) and comparable lung cancer characteristics. The PRS’s association with lung cancer mortality was validated in an unscreened cohort from the UK Biobank (*p* = 0.002). **Conclusions:** In this biomarker-based cohort study, an elevated PRS was independently associated with dying from lung cancer in both screening and non-screening cohorts.

## 1. Introduction

Lung cancer results mainly from the combined effect of genetic susceptibility and exposure to smoking or other aero-pollutants [1]. Epidemiological studies have identified a number of clinical risk variables underlying lung cancer susceptibility, which have been combined in models such as that derived from the Prostate, Lung, Colorectal and Ovarian Cancer Screening Trial (PLCO_M2012_) to estimate the risk for future lung cancer [2]. These risk models have been validated in independent cohorts to a variable degree and for performance characteristics [3]. Studies have identified single-nucleotide polymorphisms (SNPs) that are consistently associated with lung cancer, although some methodological issues exist in these studies [1,4,5]. This includes a cross-sectional study design with the potential for confounding effects and survival bias [6,7]. None of these genetic studies have validated their risk SNPs in screening populations until recently [8]. It is generally accepted that among ever-smokers, the presence of chronic obstructive pulmonary disease (COPD) is highly relevant to susceptibility to lung cancer, independent of age and smoking exposure [8,9]. We and others show that the genetic susceptibility to lung cancer among ever-smokers includes genes that also confer a risk of COPD [9,10,11,12,13]. This might explain in part the association between COPD and lung cancer [4,8].

Through candidate gene and genome-wide association studies (GWASs), several well-replicated associations between SNP variants and lung cancer have been reported [1,5]. More recently, there has been a growing interest in combining germline genetic data to derive personalised composite risk scores (termed a polygenic risk score or PRS) [14]. These risk scores are generally based on simple algorithms combining allele or genotype counts according to genotyping results and have been reported to correlate with breast and prostate cancer outcomes [15,16]. We and others have reported on PRSs for lung cancer from case–control studies [17,18] although none to date have been tested in a prospective cohort study fully representative of high-risk smokers in the community. PRSs might inform clinical practice, including targeted screening or preventive strategies for those at higher-than-average risk [14]. One aspect of this approach is the ability of the PRS to predict the behaviour or lethality of a cancer and thus the basis for more or less aggressive surveillance [16].

We have suggested that gene-based risk assessment may have clinical applicability in the setting of lung cancer screening [4,19,20]. This is because the outcomes of screening result from a complex interplay between the characteristics of the screening participant (e.g., age, comorbid disease and life expectancy) and their lung cancer biology (e.g., histology, stage and volume doubling time) [20]. The current study aimed to examine the application of a validated 12 SNP panel, selected according to their consistent replication in studies by us and others [1,5,8,9,10,11,12,13,17,18,21], to determine the panel’s effect on lung cancer risk and mortality in the context of a lung cancer screening trial. The preliminary results of this study have been reported in abstract form [21]. The effect of the 12-SNP PRS on lung cancer mortality was explored in a validation study of ever-smokers from the UK Biobank overall and in subgroups meeting the National Lung Screening Trial (NLST) and United States Preventive Services Task Force—2021 criteria for lung cancer screening.

## 2. Methods

### 2.1. Subjects

This is a secondary data analysis of the NLST. The recruitment and study design of this trial, involving 53,452 screening participants yielding 2058 histology-confirmed lung cancers, has been described elsewhere [22]. In the American College of Radiology Imaging Network (ACRIN) sub-cohort of the NLST, participants from 23 US-based centres agreed to undergo a baseline pre-bronchodilator spirometry and blood sampling for biomarker analysis (N = 10,054) (Supplementary Figure S1). Demographic data for this representative sample, including a history of pre-morbid disease, were collected through an extensive questionnaire and are summarised in Supplementary Table S1. From the total group (all ethnicities), we analysed genomic data for non-Hispanic whites comprising 9367 high-risk smokers, from which 380 lung cancers were diagnosed during the study follow-up (Supplementary Figure S1). In the NLST-ACRIN sub-study, pre-bronchodilator spirometry was measured at baseline screening (T0) in the majority of participants according to a previously published protocol [8,22]. The severity of airflow limitation was defined according to the Global Initiative on Chronic Obstructive Lung Disease (GOLD) criteria, grades 1–4 (www.GOLD.org, accessed 2 March 2021). In a validation cohort from the UK Biobank (N = 167,796 white ever-smokers), including subgroups meeting USPSTF-2021 and -NLST eligibility criteria, the 12-SNP PRS’s effect on lung cancer mortality was re-examined (imputed data).

### 2.2. Lung Cancer Outcomes

The lung cancer cases included all those diagnosed during the NLST (N = 380), whether screening- or non-screening-detected (interval), or prevalent (diagnosed at T0 or during the first year) or incident lung cancers (diagnosed during subsequent years T1 to T6) or at post-mortem [22]. All lung cancer cases were confirmed upon histological sampling according to accepted international classification criteria. Lung function results and mortality outcomes were available for all 380 lung cancer cases (100% of total). Cause of death was ascertained through a review of clinical records and death certification. Our primary aim was to examine the relationship between our PRS and the lung cancer rate (per 1000) and lung cancer deaths per 1000 screened (lung cancer mortality), independent of screening [21]. We also examined the percentage of all lung cancer deaths/all deaths and, in a secondary analysis, the percentage of lung cancer deaths/all lung cancers (lethality) before and after screening (lung cancer deaths averted with CT relative to CXR, CXR-CT) to specifically explore the PRS’s effects on screening-related outcomes.

### 2.3. Genotyping and Algorithms

Genomic DNA was extracted from buffy coat samples using standard salt-based methods, and purified genomic DNA was aliquoted (10 ng·µL^−1^ concentration) into 384-well plates. The DNA concentration and purity were determined using Nanodrop spectrophotometry. Genotyping entailed using the Sequenom^TM^ system (Sequenom^TM^ Autoflex Mass Spectrometer and Samsung 24 pin nanodispenser) by Agena (Agena BioScience, San Diego, CA, USA) multiplexed into 2 assays (Agena MassARRAY Assay Design 3.0). The Sequenom^TM^ sequences were designed in-house by Agena, with amplification and separation methods (iPLEX™, www.sequenom.com) as previously described (8). Based on prior studies in the literature, the risk genotype/s were pre-specified and assigned as susceptible (odds > 1.0) or protective (odds < 1.0) according to reported findings [17]. A call rate for each SNP of >98% was achieved. Replication using this method was undertaken in 2000 random samples and achieved a 99% accuracy for genotyping across the 12 SNPs. Using our pre-specified algorithm [17,21], the sum of the 6 susceptible genotypes (+1 for each) was added to the 6 protective genotypes (−1 for each), deriving the PRS (range −4 to +6). Genotyping and PRS were assigned blind to clinical and screening outcomes (Supplementary Tables S2 and S3). Genotyping in the UK Biobank cohort has been described elsewhere (https://www.ukbiobank.ac.uk/enable-your-research/about-our-data/genetic-data), URL accessed on 5 July 2022.

### 2.4. Statistical Analysis

Differences between groups for continuous variables were sought using an analysis of variance. Significant differences were further explored as appropriate using Tukey’s post hoc method. Differences between groups for categorical variables were sought using the chi-squared test (or exact methods where appropriate). Multivariable logistic regression (PRS as a continuous variable or grouped into tertiles) and general linear modelling (binary distribution, logit link function) were used to estimate the odds of lung cancer death, with and without an adjustment for clinical risk variables [2]. Mid-P Exact risk and rate differences were used to assess the magnitude and direction of the association (www.openpepi.com, accessed 25 March 2021). Lastly, the role of the PRS in predicting the risk of lung cancer was assessed using an area-under-the-curve analysis and a comparison made with an existing risk model [2]. Statistical significance was defined as a two-tailed *p* < 0.05. No adjustment for multiplicity was performed. All planned comparisons are presented in the tables of this paper. All analyses were performed using SAS (V 9.4, SAS Institute Inc., Cary, NC, USA), STATA v10.

## 3. Results

Based on the genotype results from 12 independent risk genotypes, we calculated a PRS for each participant (N = 9191) and sub grouped them according to low (scores −4 to −1, predominantly protective genotypes), intermediate (neutral score of zero, equal protective and susceptible genotypes) and high (scores 1 to 6, predominantly susceptible genotypes) that encompassed 31% (N = 2813), 27% (N = 2504) and 42% of the subjects (N = 3874), respectively (crude tertiles). The demographic data for these PRS subgroups are summarised in Table 1. The groups were not significantly different for most of the baseline demographic variables associated with a risk of lung cancer, specifically, age, sex, smoking status, years smoked, years quit, family history of lung cancer, self-reported history of COPD, BMI, education and pre-existing comorbidities. An increasing PRS was associated with a slightly greater cigarettes per day and pack-years, but the differences across these risk groups were small (1 cigarette/day and 1 pack year). Despite a comparable lung function, a higher PRS was associated with a slightly greater spirometry-defined COPD (GOLD grades 1–4), although again the differences were very small (33% vs. 36%, *p* = 0.02) (Table 1).

**Figure 1:**
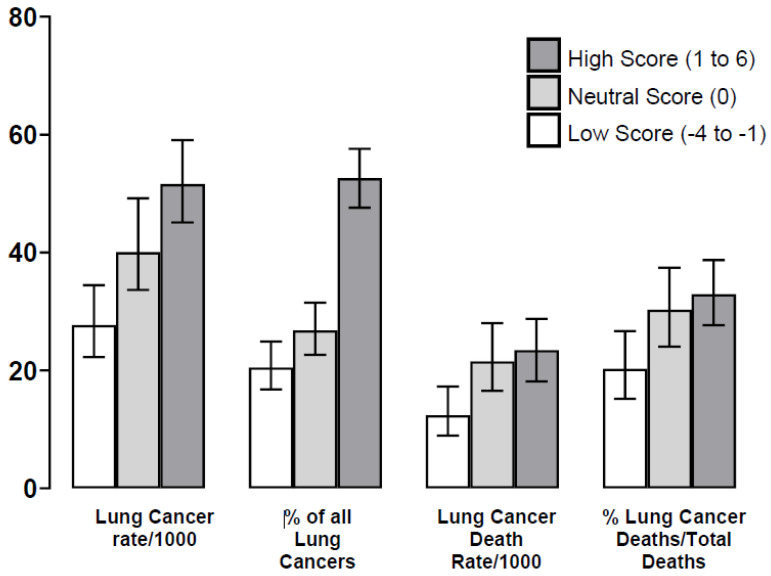
Outcomes for lung cancer according to the lung cancer polygenic risk score (PRS) groups (Table 2). **Legend**: The lung cancer death rate according to PRS category (low, neutral and high) is significantly different (*p* = 0.004) and may in part reflect lung cancer incidence or risk (*p* < 0.0001). However, we found that, despite similar demographics, there were significant differences in the %lung cancer deaths/total deaths according to these subgroups (*p* = 0.0012), and relative to a low score (19%), death rates were 30% and 33% for those with a neutral and high score, respectively (*p* = 0.010 and *p* = 0.0006, respectively). This may indicate an independent effect on lung cancer biology toward greater aggressivity in those with an elevated (neutral or high) PRS.

**Before randomisation by screening arm**: An increasing PRS was associated with a linear increase in lung cancer incidence (*p* < 0.0001), absolute lung cancer deaths (*p* = 0.0040) (Table 2, Supplementary Table S2 and S3) and lung cancer deaths as a proportion of total deaths (*p* = 0.0012) (Table 2 and Figure 1). Notably, subjects in the intermediate (neutral) and high-risk PRS subgroups had a similar lung cancer mortality (mortality rate ratios, Table 2), which were 1.7- and 1.9-fold greater, respectively (*p* = 0.01 and *p* = 0.0009), than those in the low-PRS group (Table 2, Figure 1). These findings were evident, despite a very similar lung function, histology, stage, detection method, surgical rates and diagnostic screening interval (Supplementary Table S2). When we compared cause-specific deaths according to an increasing PRS, only lung cancer mortality demonstrated a positive relationship (Table 2, Supplementary Figure S2). After adjustment for various clinical variables, we found that lung cancer mortality remained significantly greater in the high- versus low-PRS groups (Supplementary Figure S3). PRS, as a continuous variable, remained an independent predictor of lung cancer death after extensive adjustment in our multivariable model (reduced model *p* = 0.0027, Table 3). In our secondary analysis the lethality was 47% and 41% for an elevated PRS (neutral/high) and a low PRS, respectively (*p* = 0.40). In the UK Biobank sample of 167,796 ever-smokers, the PRS was again independently associated with dying from lung cancer (*p* = 0.002), albeit with a reduced odds ratio (OR = 1.06, 95% CI 1.02–1.10) (Supplementary Table S4a). In substantially smaller sub-cohorts, meeting the USPSTF 2021 criteria (N = 34,356) and NLST-eligible criteria (N = 20,796), the magnitude of the PRS association with lung cancer mortality (OR) was unchanged, albeit no longer statistically significant at a 5% threshold with smaller sample sizes (Supplementary Tables S4 and S5).

**Figure 2:**
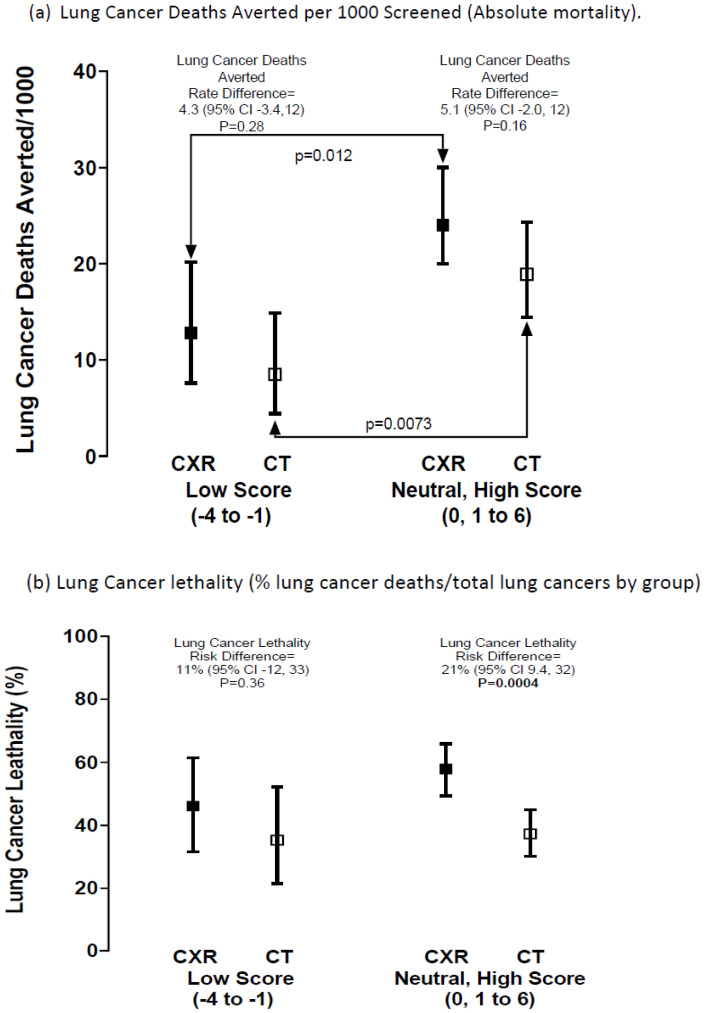
Risk difference for (**a**) lung cancer deaths averted per 1000 screened and (**b**) lung cancer lethality, according to PRS risk grouping after screening randomisation (Table 2). **Legend:** Comparing the lung cancers death rate/1000 screened according to screening arm (Figure 2a); for a low v elevated (neutral–high) PRS score in the CXR arm (*p* = 0.012) and for those in the CT arm (*p* = 0.0073). This indicates that the greater lung cancer mortality in those with an elevated versus low PRS is independent of screening arm.

**After randomisation by lung cancer screening arm (CT vs. CXR)**: After stratification by screening arm, participants in the elevated (neutral–high) PRS group had a greater lung cancer death rate/1000 screened compared to a low PRS (CT arm *p* = 0.0073 and CXR *p* = 0.012) (Figure 2a). Those in the elevated-PRS group randomised to CXR had the greatest lung cancer lethality (21% higher than CT, *p* = 0.0004, Table 2, Figure 2b). Those in the elevated-PRS group also had a greater odds ratio (OR) of lung cancer death in the CXR arm compared to the CT arm (37% vs. 58%, OR = 2.32, 95% CI 1.15–3.70, *p* = 0.0005), which was a difference not observed in the low-PRS group (35% vs. 46%, OR = 1.57, 95% CI 0.61–4.04, *p* = 0.36) (Table 2, Figure 2b). This was in conjunction with comparable surgical rates favouring those randomised to CT screening over CXR (15%, *p* = 0.21 and 14%, *p* = 0.019 for low and elevated PRS groups, respectively) and comparable lung cancer characteristics (Supplementary Table S3). However, while the risk difference for lung cancer deaths averted after randomisation was not significantly different between CT and CXR, according to elevated- and low-PRS subgroups (21% vs. 34% respectively), the screening appeared marginally more efficient in the elevated-PRS group (5.1 vs. 4.3 lung cancer deaths averted per 1000 screened), where lung cancer accounted for >30% of all deaths compared to only 19% in the low-PRS group (*p* = 0.0012) (Table 2).

**Discriminative performance**: We found that our model, using PRS alone to predict the risk of dying of lung cancer, had an area under the receiver operating curve (AUC) of 0.568 (95% confidence interval (CI) = 0.528–0.608, *p* = 0.0009 relative to chance); and according to the PRS plus clinical score (composite gene-based score), an AUC = 0.661 (95% CI = 0.623–0.698, *p* < 0.0001 relative to the PRS alone). Using the same analysis for the risk of developing lung cancer, we found for the clinical score alone an AUC = 0.647 (95%CI 0.620–0.674, *p* < 0.0001 relative to chance), and adding the PRS score to the clinical score increased the AUC (AUC = 0.667, 95% CI 0.640–0.693, *p* = 0.018 compared to the clinical score alone). When this was limited to those in the CT arm, the clinical-only AUC (0.640, 95% CI 0.602–0.678) increased when adding the PRS (0.668, 95% CI 0.602–0.705, *p* = 0.011). Using the PLCO_M2012_ model for developing lung cancer in this cohort (2), we found an AUC = 0.677 (95% CI 0.651–0.704, *p* < 0.0001) relative to chance. The 6-year risk of developing lung cancer based on our gene-based model was linear and compared favourably with the PLCO_M2012_ model in this representative sample of the NLST (Supplementary Table S1 and Supplementary Figure S4). For the UK Biobank analysis meeting NLST criteria (N = 20,796), the 6-year risk of developing lung cancer in the composite score had an AUC = 0.628 (95% CI 0.607–0.648), not quite overlapping to that derived using the PLCO_M2012_ (AUC = 0.675, 95% CI 0.654–0.694). The AUC for the clinical score alone (0.627 95% CI 0.602–0.634) increased marginally with the inclusion of the PRS (AUC for PRS alone = 0.511, 95% CI 0.500–0.528) (Supplementary Table S5).

## 4. Discussion

In this biomarker-based study in a subgroup of the NLST, we report that a lung cancer polygenic risk score (PRS), calculated from 12 independent risk genotypes, appears to correlate with lung cancer mortality. The PRS was based on germline mutations and is independent of lung cancer risk factors, notably, age, smoking, family history, education, BMI, lung function and pre-existing comorbidity. While we expected there to be more cancers developing in those with the higher PRS scores, we were surprised to find that an elevated PRS was also significantly and linearly associated with greater lung cancer deaths. This could not be explained by a variation in lung cancer stage, histology, detection methods, screening interval or surgical rates. Although there was a weak association with smoking pack-years across the PRS groups, due primarily to smoking one extra cigarette per day, this would not explain our findings. Similarly, there was marginally more airflow limitation in those with an elevated PRS, but neither this, nor any other clinical variables examined after extensive adjustment, could account for the persisting effect between PRS and lung cancer mortality in our multivariable model. While we note our PRS was independently associated with lung cancer mortality (but not other causes of death), the basis of this remains unclear. After stratification by screening arm, a significant reduction in lung cancer lethality in the CT arm was found in those with an elevated score but not a low score (a 21% versus 11% reduction, respectively). The association with lung cancer mortality was validated, after adjustments including age, smoking and lung function, in an unscreened cohort of ever-smokers from the UK Biobank. We conclude that our lung cancer PRS appears to provide an independent and specific biomarker of lung cancer death in the setting of a screening study and, on this basis, may contribute to optimising screening outcomes [23].

There has been considerable interest in assessing high-risk smokers eligible for lung cancer screening using clinical risk models [24]. The most validated of these risk models is the PLCO_M2012_, which combines a number of clinical risk variables to derive a 6-year risk for developing lung cancer [2]. However, we have shown that the PLCO_M2012_ also predicts the likelihood of having an airflow limitation and COPD [20,25]. This is important, because pre-existing COPD is associated with a greater lung cancer risk, more aggressive lung cancer histological subtypes, less surgery, more comorbidity with increasing non-lung cancer deaths and a reduced benefit of lung cancer screening [20]. This means those at the greatest risk of lung cancer may not achieve the greatest benefits from screening due to an attenuation of this risk–benefit relationship at the highest end of the risk spectrum [20,26]. As the benefits of lung cancer screening result from a complex interplay between a number of variables [20], it might be helpful to identify a biomarker of lung cancer mortality as part of the general risk assessment of screening participants [20,23,27]. This is because it is recognised there exists considerable biological variability between lung cancers and that this variability will affect outcomes following screening [28]. For example, for elderly participants with a high PRS and high clinical risk score with severe COPD, the harms of screening may outweigh the benefits and attenuate the efficiency of screening [29]. Similarly, those with a low PRS with a low clinical score and negative CT may defer screening or screen two-yearly. Alternatively, an elevated PRS may be used as a motivational tool for screening adherence and smoking cessation. We propose that a biomarker of lung cancer biology could be combined with clinical risk variables to improve screening outcomes (participation or adherence) and better individualise the risk versus benefits of screening.

The application of adding genetic data to clinical risk scores for cancer has been gaining support in the clinical setting of optimising screening for prostate and breast cancer [15,16]. Investigators have previously shown that SNP-based data add a small but clinically meaningful contribution to risk-stratifying high-risk populations. In this setting, we have shown in the current study that our PRS, based on previously validated SNPs, adds to the risk assessment for lung cancer in a lung cancer screening cohort where lung cancer was diagnosed prospectively. In the current analysis, we show that while the PRS is associated with a small increase in the risk of developing lung cancer (it improves the AUC when added to our clinical score), it is associated with a 1.7–1.9-fold greater mortality from lung cancer. This latter finding persisted after extensive adjustment for various clinical variables and was also independent of known lung cancer variables such as stage and histology.

This study has strengths and limitations. The modest number of lung cancers reflects the challenge of prospective studies of lung cancer. The full NLST enrolled over 53,000 high-risk smokers and identified about 2000 lung cancer cases across the two screening arms [22]. In our study, we have just under one-fifth of the original 50,000. While further validation studies are necessary [27,29], only a limited number of randomised control trials have collected biomarker specimens for clinical validation [22]. Another limitation of this study is the use of specific SNP variants, albeit highly replicated in several large cohorts, with an associated biological plausibility [10,11,12,30,31,32,33,34,35,36,37,38,39,40,41,42,43,44,45,46,47,48,49,50,51,52,53,54,55,56,57,58,59]. In the future, entire genetic regions may be sequenced to better refine risk. The further limitations of this study are that the PRS relates to non-Hispanic whites only, the interactions between SNPs (epigenetic effects) are unknown, and residual confounding from non-genetic factors remains a possibility. These limitations are shared with all SNP-based polygenic risk scores and may be better understood with larger data sets and advances in analytical approaches [60,61,62]. The strengths of this study include statistical evidence of the clinical validity of the PRS in a highly representative, albeit older, screening-eligible population (55–74 yo with 30+ pack-years) [27,30]; genotyping was performed blind to screening outcomes and targeted well-validated SNPs; and the algorithm combining risk genotypes made no assumptions about effect size or gene–gene interaction (to minimise over-fitting). The current study is part of an ongoing validation (not model building), where we have used pre-specified risk genotypes and a pre-specified algorithmic approach to calculate the PRS [17,21]. In this proof-of-concept study, after extensive adjustment the PRS was again independently associated with dying of lung cancer in a subgroup of the UK Biobank, an unscreened cohort matched for ethnicity but distinctly different lung cancer risk profiles (Supplementary Table S1). The PRS was also associated with lung cancer mortality in those meeting USPSTF-2021 eligibility. Interestingly, we found the time to lung cancer diagnosis was shorter in the high-PRS group (data not shown). Lastly, our PRS was correlated with those who died of their lung cancer, not just those who developed lung cancer, as reported by retrospective case–control studies [1,5]. A novel approach in this study was the inclusion of genetic factors underlying both lung cancer and COPD. We note the PRS did correlate weakly with smoking intensity and COPD but not severity of airflow limitation. These represent potentially confounding (or mediating) effects that have to date been overlooked when examining the genetic basis of lung cancer [1,4,5].

In summary, we have calculated a polygenic risk score for lung cancer that appears to independently correlate with lung cancer incidence and mortality in a prospective clinical validity study [27,29]. This PRS was also associated with greater mortality specifically for lung cancer and regardless of the screening arm. We propose, subject to further validation, that this PRS may represent a marker of lung cancer biology. As a PRS represents a very stable, cheap, and easily accessible bioassay, it could be incorporated into the optimisation of screening programmes. Specifically, it could help contribute to assessing lung cancer screening participants with regards to the benefits of screening at an individualised level (clinical utility) [20,29].

## Figures and Tables

**Table 1:** Lung cancer risk factors and demographic variables according to 12-SNP polygenic risk score (PRS) severity in the NLST-ACRIN sub-study (N = 9191).

Polygenic Risk Score Groups (Absolute Score Range)	Low Score (−4 to −1)	Neutral Score 0	High Score (1 to 6)	*p* Value
N = 9191 (% total) NHW	2813 (30.6%)	2504 (27.2%)	3874 (42.1%)	
**Demographics**				
Age—mean (SD)	61.7 (5.2)	61.9 (5.1)	61.8 (5.1)	0.40
Sex—% male	1608 (57.2%)	1428 (57.0%)	2190 (56.5%)	0.86
Smoking status—current smoker	1374 (48.8%)	1161 (46.4%)	1882 (48.6%)	0.14
Pack-years—mean (SD)	55.2 (22.7)	55.7 (23.2)	57.1 (23.7)	**0.0021**
Cigarettes/day—mean (SD)	27.7 (10.8)	28.0 (10.9)	28.7 (11.3)	**0.0002**
Years quit mean (SD)	3.6 (5.0)	3.8 (5.1)	3.8 (5.1)	0.40
Smoking duration years mean (SD)	40.3 (7.1)	40.4 (7.5)	40.3 (7.6)	0.93
Family history of lung cancer—yes (%)	671 (23.9%)	607 (24.2%)	912 (23.5%)	0.81
Self-reported Hx of COPD (%) ‡	561 (19.9%)	508 (20.3%)	819 (21.1%)	0.46
BMI mean (SD)	27.8 (5.0)	27.9 (5.2)	27.9 (5.1)	0.87
Education Level—N (%)				
-High school or less	809 (28.8%)	677 (27.0%)	1138 (29.4%)	
-Post high School/some college	995 (35.4%)	912 (36.4%)	1359 (35.1%)	
-College graduate	508 (18.1%)	403 (16.1%)	661 (17.1%)	0.05
-Postgraduate/professional	423 (15.0%)	453 (18.1%)	617 (15.9%)	
-Other/unknown	78 (2.8%)	59 (2.4%)	99 (2.6%)	
**Lung Function**				
FEV_1_/FVC—mean (SD)	71.4 (10.7)	70.93 (10.9)	70.8 (10.6)	0.057
FEV_1_% predicted—mean (SD)	81.9 (19.4)	81.4 (20.5)	80.9 (20.2)	0.15
Total COPD GOLD 1–4	925 (32.9%)	895 (35.7%)	1394 (36.0%)	**0.02**
**Pre-morbid conditions (self-report)**				
COPD	182 (6.5%)	195 (7.8%)	285 (7.4%)	0.16
Chronic bronchitis	327 (11.6%)	254 (10.1%)	442 (11.5%)	0.18
Emphysema	232 (8.2%)	237 (9.5%)	378 (9.8%)	0.10
Asthma—adult	181 (6.4%)	171 (6.8%)	272 (7.0%)	0.60
Pneumonia	772 (27.4%)	709 (28.3%)	1099 (28.4%)	0.67
Heart disease	392 (13.9%)	312 (12.5%)	521 (13.4%)	0.28
Hypertension	1002 (35.6%)	890 (35.5%)	1374 (35.5%)	0.99
Stroke	91 (3.2%)	66 (2.6%)	109 (2.8%)	0.40
Diabetes	243 (8.6%)	251 (10.0%)	342 (8.8%)	0.16
Any cancer history	121 (4.3%)	97 (3.9%)	163 (4.2%)	0.71

‡ Self-reported history of COPD = “Has a doctor ever told you that you have any of the following conditions?” COPD, emphysema, chronic bronchitis or adult asthma (where prior childhood asthma is excluded). COPD = chronic obstructive pulmonary disease. *p* < 0.05 are bolded.

**Table 2:** Overall mortality and reduction in lung cancer deaths after screening according to polygenic risk score (PRS) groups (low, neutral or high) in the NLST-ACRIN sub-study (N = 9191).

Polygenic Risk Score Groups (Absolute Score Range)	Low Score (−4 to −1)	Neutral Score 0	High Score (1 to 6)
N = 9191 NHW (% of total cohort)	2813 (30.6%)	2504 (27.2%)	3874 (42.1%)
**Lung Cancer (LC)** (% total, N = 380) ^‡^	78 (20.5%)	102 (26.8%)	200 (52.6%)
Incidence/per 1000 (95% CI)	27.7 (22.3,34.5)	40.7 (33.7,49.2)	51.6 (45.1,59.1) ^1^
**Overall Mortality (prevalence)**			
Total Deaths (%, per 100)	187 (6.6%)	178 (7.1%)	276 (7.1%)
% and 95% CI all deaths (N = 641)	29.2%, (25.8, 32.9)	27.8% (24.4, 31.4)	43.1% (39.3, 46.9)
-LC Deaths (% total deaths by group)	35/187 (18.7%)	54/178 (30.3%)	91/276 (33.0%) ^2^
-% all deaths (N = 641)	5.5% (4.0, 7.5)	8.4% (6.5, 10.8)	14.2% (11.7, 17.1)
-% all deaths by tertile	1.2% (0.90, 1.73)	2.2% (1.7, 2.8)	2.3% (1.9, 2.9) ^3^
-Cardiovascular (CVD) Deaths	41 (21.9%)	40 (22.5%)	63 (22.8%)
-% all deaths (N = 641)	6.4% (4.8, 8.6)	6.2% (0.2,1.6)	9.8% (7.8, 12.4)
-% all deaths by tertile	1.5% (1.1, 2.0)	1.6% (1.2, 2.2)	1.6% (1.3, 2.1)
-Respiratory Deaths	22 (11.8%)	16 (9.0%)	19 (6.9%)
-% all deaths (N = 641)	3.4% (2.3, 5.1)	2.5% (1.5, 4.0)	3.0% (1.9, 4.6)
-% all deaths by tertile	0.78% (0.52, 1.18)	0.64% (0.39, 1.04)	0.49% (0.31, 0.76)
-Other Cancer Deaths	42 (22.5%)	41 (23.0%)	56 (20.3%)
-% all deaths (N = 641)	6.6% (4.9, 8.7)	6.4% (4.8, 8.6)	8.7% (6.8, 11.2)
-% all deaths by tertile	1.5% (1.1, 2.0)	1.6% (1.2, 2.2)	1.4% (1.1, 1.9)
-Other Deaths or Cause Unknown	47 (25.1%)	27 (15.2%)	47 (17.0%)
-% all deaths (N = 641)	7.3% (5.6, 9.6)	4.2% (2.9, 6.1)	7.3% (5.6, 9.6)
-% all deaths by tertile	1.7% (1.3, 2.2)	1.1% (0.7, 1.6)	1.2%(0.9, 1.6)
**Lung Cancer Mortality—rate ratios ^‡^**			
Low vs. Neutral	ref	1.73	1.89
(95% CI), *p* value		(1.14–2.64), ***p* = 0.010**	(1.28–2.78), ***p* = 0.0009**
Neutral vs. High	-	ref	1.09
(95% CI), *p* value			(0.78–1.52), *p* = 0.62
**Lung Cancer Screening Outcomes**			
Outcomes Low vs. Neutral + High	Low—CXR vs. CT	Neutral + High—CXR vs. CT	
Surgery rates % (95% CI) favouring CT over CXR for PRS subgroup	17/39 vs. 20/34	61/133 vs. 96/161	
	44% (29, 59) vs. 59% (42, 74)	46% (38, 54) vs. 60% (52, 67)	
Risk difference—% (95% CI)	−15% (−38, 7.5)	−14% (−25, −2.41)	
*p* value	*p* = 0.21	***p* = 0.019**	
Lung Cancer (LC) Lethality	Low—CXR vs. CT	Neutral + High—CXR vs. CT	
LC lethality in CXR vs. CT ^‡‡^	18/39 vs. 12/34	77/133 vs. 60/161	
% (95% CI)	46% (32, 61) vs. 35% (21, 52)	58% (49, 66) vs. 37% (30, 45)	
Risk difference—% difference (95% CI)	+11.0 (−12, 33)	+21% (9.4,32)	
*p* value	*p* = 0.36	***p* = 0.0004**	
Odds ratio (OR) of LC lethality in CXR vs. CT (95% CI)			
	OR = 1.57 (0.61, 4.04)	OR = 2.32 (1.15, 3.70)	
*p* value	*p* = 0.35	***p* = 0.0005**	
Lung Cancer Deaths Averted by Screening (CXR-CT)			
Deaths averted CT versus CXR	18/1406 vs. 12/1405	77/3204 vs. 60/3174	
Absolute difference/1000 screened (% relative reduction)	−4.3 (−3.4, 12)	−5.1 (−2.0, 12)	
	34% relative reduction	21% relative reduction	
Odds ratio (OR) of LC deaths averted in CXR vs. CT (95% CI)			
	OR = 0.66 (−0.31, 1.38)	OR = 0.79 (0.56, 1.10)	
*p* value	*p* = 0.28	*p* = 0.16	

^‡^ Includes lung cancer deaths identified at post-mortem (N = 180), ^‡‡^ Only lung cancers diagnosed during screening and follow-up (N = 367). *p* value according to PRS group. (95% CI) = 95% confidence interval. *p* < 0.05 is bold. *p* < 0.0001 ^1^, *p* = 0.0012 ^2^, *p* = 0.0040 ^3^.

**Table 3:** Multivariable logistic regression predicting lung cancer death according to known risk variables, including PRS (treated as a continuous variable), in the NLST-ACRIN sub-study (N = 9191).

Variable	Full Model			Reduced Model *		
	Point Estimate	95% CI	*p* Value	Point Estimate	95% CI	*p* Value
Age	1.07	1.04–1.10	<0.0001	1.07	1.03–1.10	<0.0001
Sex (male vs. female)	0.98	0.67–1.38	0.89			
Current smoker	1.69	1.19–2.40	0.0031	1.69	1.19–2.39	0.0033
Pack-years	1.01	1.01–1.02	0.0004	1.01	1.01–1.02	0.0002
Family history of LC	1.28	0.89–1.85	0.18			
Self-reported COPD	1.40	0.97–2.03	0.070	1.41	0.98–2.02	0.07
BMI	0.95	0.91–0.99	0.0076	0.95	0.92–0.99	0.0073
High school/some college	1.49	1.03–2.16	0.035	1.52	1.05–2.19	0.027
12-SNP lung cancer PRS	1.18	1.06–1.32	0.0026	1.18	1.06–1.32	0.0027
COPD (GOLD 1–4) ^#^	1.45	1.03–2.06	0.036	1.47	1.04–2.09	0.029
Screening arm (CXR vs. CT)	1.23	0.88–1.71	0.22			

^#^ COPD (GOLD 1–4) = airflow limitation upon pre-bronchodilator spirometry. * Reduced model where variables with *p* > 0.15 were removed. LC = lung cancer. PRS = polygenic risk score. BMI = body mass index. CXR = chest X-ray, CT = computed tomography. **Legend**: Given the marginal increase across the PRS tertiles in Table 1 for smoking (pack-years and cigs/day) and COPD (GOLD 1–4) status, an extensive adjustment for all these risk variables was undertaken.

## Data Availability

No new data was created during this study, but data is available with application to the American College of Radiology Imaging Network, fduan@stat.brown.edu.

## Supplementary Materials

The following supporting information can be downloaded at: https://www.mdpi.com/article/10.3390/jcm14093110/s1, Figure S1: Consort figure of the genetic study subgroup from the NLST; Figure S2: Cause-specific mortality analysed across PRS groups as (a) percentage of all deaths and (b) deaths per 1000 within each PRS risk group by crude tertiles; Figure S3: Odds of lung cancer deaths referenced against the low PRS risk group and adjusted according to clinical factors, clinical score, and GOLD 1–4 status; Figure S4: Lung cancer prevalence per 1000 using the gene-based (blue), and PLCOM2012 (red) models according to quintiles of 6-year risk of developing lung cancer Table S1: Comparison of the ACRIN sub-cohort (N = 9191), CT arm of the NLST (N = 26,723) and NLST-eligible UK Biobank sub-study (N = 20,796), according to clinical variable; Table S2: Lung cancer (LC) characteristics according to the lung cancer polygenic risk score (PRS) score (Low, Neutral, High; Table S3: Algorithm and referencing for the polygenic risk score (PRS) and clinical score for lung cancer; Table S4: Multivariable logistic regression predicting lung cancer death according to known risk variables including PRS (treated as a continuous variable) in the UK Biobank of ever smokers and those according to USPSTF and NLST screening criteria; Table S5: Area-under-the curve analyses for 6-year risk of developing or dying of lung cancer in the NLST and UK biobank (validation) sub-groups according to the gene-based composite (PRS + Clinical) score and PLCOM2012 model.

## References

[1] Brennan P., Hainaut P., Bofetta P. (2011). Genetics of lung-cancer susceptibility. Lancet Oncol..

[2] Tammemägi M.C., Katki H.A., Hocking W.G., Church T.R., Caporaso N., Kvale P.A., Chaturvedi A.K., Silvestri G.A., Riley T.L., Commins J. (2013). Selection Criteria for Lung-Cancer Screening. N. Engl. J. Med..

[3] ten Haaf K., Jeon J., Tammemagi M.C., Han S.S., Kong C.Y., Plevritis S.K., Feurer E.J., de Koning H.J., Steyerberg E.W., Meza R. (2017). Risk prediction models for selection of lung cancer screening candidates: A retrospective validation study. PLoS Med..

[4] El-Zein R.A., Young R.P., Hopkins R.J., Etzel C.J. (2012). Genetic Predisposition to Chronic Obstructive Pulmonary Disease and/or Lung Cancer: Important Considerations When Evaluating Risk. Cancer Prev. Res..

[5] Bosse Y., Amos C.I. (2018). A decade of GWAS results in lung cancer. Cancer Epidemiol. Biomark. Prev..

[6] Schulz K.F., Grimes A.D. (2002). Case-control studies: Research in reverse. Lancet.

[7] Hu Z.-H., Connett J.E., Yuan J.-M., Anderson K.E. (2016). Role of survivor bias in pancreatic cancer case-control studies. Ann. Epidemiol..

[8] Hopkins R.J., Duan F., Gamble G.D., Chiles C., Cavadino A., Billings P., Aberle D., Young R.P. (2021). Chr15q25 genetic variants (rs 16969968) independently confers risk of lung cancer, COPD and smoking intensity in a prospective study of high-risk smokers. Thorax.

[9] Young R.P., Hopkins R.J., Hay B.A., Epton M.J., Black P.N., Gamble G.D. (2008). Lung cancer gene associated with COPD: Triple whammy or possible confounding effect?. Eur. Respir. J..

[10] Lambrechts D., Buysschaert I., Zanen P., Coolen J., Lays N., Cuppens H., Groen H.J.M., van Klaveren R.J., Vershakelen J., Dewever W. (2010). The 15q24/25 susceptibility variant for lung cancer and chronic obstructive pulmonary disease is associated with emphysema. Am. J. Respir. Crit. Care Med..

[11] Wauters E., Smeets D., Coolen J., Vershakelen J., Leyn P., Decramer M., Vansteenkiste J., Janssens W., Lambrechts D. (2011). The TERT-CLPTM1L locus for lung cancer predisposes to bronchial obstruction and emphysema. Eur. Respir. J..

[12] Gabrielsen M.E., Romundstad P., Langhammer A., Krokan H.E., Skorpen F. (2013). Association between a 15q25 gene variant, nicotine-related habits, lung cancer and COPD among 56 307 individuals from the HUNT study in Norway. Eur. J. Hum. Genet..

[13] Ziółkowska-Suchanek I., Mosor M., Gabryel P., Grabicki M., Żurawek M., Fichna M., Strauss E., Batura-Gabryel H., Dyszkiewicz W., Nowak J. (2015). Susceptibility loci in lung cancer and COPD: Association of IREB2 and FAM13A with pulmonary diseases. Sci. Rep..

[14] Torkamani A., Wineinger N.E., Topol E.J. (2018). The personal and clinical utility of polygenic risk scores. Nat. Rev. Genet..

[15] Mavaddat N., Michailidou K., Dennis J., Fachal L., Lee A., Tyrer J.P., Chen T.-H., Wang Q., Bolla M.K., Yang X. (2019). Polygenic Risk Scores for Prediction of Breast Cancer and Breast Cancer Subtypes. Am. J. Hum. Genet..

[16] Seibert T.M., Fan C.C., Wang Y., Zuber V., Karunamuni R., Parsons J.K., Eeles R.A., Easton D.F., Kote-Jarai Z., Al Olama A.A. (2018). Polygenic hazard score to guide screening for aggressive prostate cancer: Development and validation in large scale cohorts. BMJ.

[17] Young R.P., Hopkins R.J., Whittington C.F., Hay B.A., Epton M.J., Gamble G.D. (2011). Individual and Cumulative Effects of GWAS Susceptibility Loci in Lung Cancer: Associations after Sub-Phenotyping for COPD. PLoS ONE.

[18] Weissfeld J.L., Lin Y., Lin H.-M., Kurland B.F., Wilson D.O., Fuhrman C.R., Pennathur A., Romkes M., Nukui T., Yuan J.-M. (2015). Lung Cancer Risk Prediction Using Common SNPs Located in GWAS-Identified Susceptibility Regions. J. Thorac. Oncol..

[19] Young R.P., Hopkins R.J., Hay B.A., Epton M.J., Mills G.D., Black P.N., Gardner H.D., Sullivan R., Gamble G.D. (2009). Lung Cancer Susceptibility Model Based on Age, Family History and Genetic Variants. PLoS ONE.

[20] Young R.P., Hopkins R.J. (2018). Chronic obstructive pulmonary disease (COPD) and lung cancer screening. Transl. Lung Cancer Res..

[21] Young R.P., Hopkins R.J., Duan F., Chiles C., Aberle D., Gamble G.D. (2018). Genetic risk score from 12 SNP panel predicts lung cancer lethality in the National Lung Screening Trial (NLST)–A validation study in the NLST_ACRIN (N = 10,054). Am. J. Respir. Crit. Care Med..

[22] Aberle D.R., Adams A.M., Berg C.D., Black W.C., Clapp J.D., Fagerstrom R.M., Gareen I.F., Gatsonis C., Marcus P.M., National Lung Screening Trial Research Team (2011). Reduced Lung-Cancer Mortality with Low-Dose Computed Tomographic Screening. N. Engl. J. Med..

[23] Vickers A.J., Sud A., Bernstein J., Houlston R. (2022). Polygenic risk scores to stratify cancer screening should predict mortality not incidence. npj Precis. Oncol..

[24] Tammemägi M.C. (2018). Selecting lung cancer screenees using risk prediction models—Where do we go from here. Transl. Lung Cancer Res..

[25] Young R.P., Hopkins R.J., Duan F., Chiles C., Aberle D., Gamble G.D. (2018). The relationship between lung cancer risk according to the PLCO2012 model and prevalence or presence of COPD in the NLST-ACRIN sub-study (N = 10,054). Am. J. Respir. Crit. Care Med..

[26] Rivera M.P., Tanner N.T., Silvestri G.A., Detterbeck F.C., Tammemägi M.C., Young R.P., Slatore C.G., Caverly T.J., Boyd C.M., Braithwaite D. (2018). Incorporating Coexisting Chronic Illness into Decisions about Patient Selection for Lung Cancer Screening. An Official American Thoracic Society Research Statement. Am. J. Respir. Crit. Care Med..

[27] Mazzone P.J., Sears C.R., Arenberg D.A., Gaga M., Gould M.K., Massion P.P., Nair V.S., Powell C.A., Silvestri G.A., Vachani A. (2017). Evaluating molecular biomarkers for the early detection of lung cancer: When is a biomarker ready for clinical use?. Am. J. Respir. Crit. Care Med..

[28] Chirieac L.R., Flieder D.B. (2010). High-Resolution Computed Tomography Screening for Lung Cancer: Unexpected Findings and New Controversies Regarding Adenocarcinogenesis. Arch. Pathol. Lab. Med..

[29] Lebrett M.B., Crosbie E.J., Smith M.J., Woodward E.R., Evans D.G., Crosbie P.A.J. (2021). Targeting lung cancer screening to individuals at greatest risk: The role of genetic factors. J. Med. Genet..

[30] Thorgeirsson T.E., Geller F., Sulem P., Rafnar T., Wiste A., Magnusson K.P., Manolescu A., Thorleifsson G., Stefansson H., Ingason A. (2008). A variant associated with nicotine dependence, lung cancer and peripheral arterial disease. Nature.

[31] Hung R.J., McKay J.D., Gaborieau V., Boffetta P., Hashibe M., Zaridze D., Mukeria A., Szeszenia-Dabrowska N., Lissowska J., Rudnai P. (2008). A susceptibility locus for lung cancer maps to nicotinic acetylcholine receptor subunit genes on 15q25. Nature.

[32] Amos C.I., Wu X., Broderick P., Gorlov I.P., Gu J., Eisen T., Dong Q., Zhang Q., Gu X., Vijayakrishnan J. (2008). Genome-wide association scan of tag SNPs identifies a susceptibility locus for lung cancer at 15q25. 1. Nat. Genet..

[33] Broderick P., Wang Y., Vijayakrishnan Matakidou A., Spitz M.R., Eisen T., Amos C.I., Houlston R.S. (2009). Deciphering the impact of common genetic variation on lung cancer risk: A genome-wide association study. Cancer Res..

[34] Wang J., Liu Q., Yuan S., Xie W., Liu Y., Xiang Y., Wu N., Wu L., Ma X., Cai T. (2017). Genetic predisposition to lung cancer: Comprehensive literature integration, meta-analysis, and multiple evidence assessment of candidate-gene association studies. Sci. Rep..

[35] Timofeeva M.N., McKay J.D., Smith G.D., Johansson M., Byrnes G.B., Chabrier A., Relton C., Ueland P.M., Vollset S.E., Midttun O. (2011). Genetic polymorphisms in 15q and 19q13 loci, cotinine levels and risk of lung cancer in EPIC. Cancer Epidemiol. Biomark. Prev..

[36] Pillai S.G., Ge D., Zhu G., Kong X., Shianna K.V., Need A.C., Feng S., Hersh C.P., Bakke P., Gulsvik A. (2009). A Genome-Wide Association Study in Chronic Obstructive Pulmonary Disease (COPD): Identification of Two Major Susceptibility Loci. PLOS Genet..

[37] Hancock D.B., Eijgelsheim M., Wilk J.B., Gharib S.A., Loehr L.R., Marciante K.D., Franceschini N., van Durme Y.M.T.A., Chen T.-H., Barr R.G. (2010). Meta-analyses of genome-wide association studies identify multiple loci associated with pulmonary function. Nat. Genet..

[38] Ragland M.F., Benway C.J., Lutz S.M., Bowler R.P., Hecker J., Hokanson J.E., Crapo J.D., Castaldi P.J., DeMeo D.L., Hersh C.P. (2019). Genetic advances in chronic obstructive pulmonary disease: Insights from COPD Gene. Am. J. Respir. Crit. Care Med..

[39] Bierut L.J. (2009). Nicotine dependence and genetic variation in the nicotinic receptors. Drug Alcohol. Depend..

[40] Janes A.C., Smoller J.W., David S.P., Frederick B.D., Haddad S., Basu A., Fava M., Evins A.E., Kaufman M.J. (2012). Association between CHRNA5 genetic variation at rs16969968 and brain reactivity to smoking images in nicotine dependent women. Drug Alcohol. Depend..

[41] Wang J., Spitz M.R., Amos C.I., Wu X., Wetter D.W., Cinciripini P.M., Shete S. (2010). Mediating effects of smoking and COPD on the relationship between CHRNA5-A3 genetic locus and lung cancer risk. Cancer.

[42] Kaur-Knudsen D., Nordestgaard B.G., Bojesen S.E. (2012). *CHRNA3*genotype, nicotine dependence, lung function and disease in the general population. Eur. Respir. J..

[43] Chong I.-W., Chang M.-Y., Chang H.-C., Yu Y.-P., Sheu C.-C., Tsai J.-R., Hung J.-Y., Chou S.-H., Tsai M.-S., Hwang J.-J. (2006). Great potential of a panel of multiple hMTH1, SPD, ITGA11, and COL11A1 markers for the diagnosis of patients with non-small cell lung cancer. Oncol. Rep..

[44] Zhu C.Q., Popova S.N., Brown E.R.S., Barsyte-Lovejoy D., Navab R., Shih W., Li M., Lu M., Jurisica I., Penn L.Z. (2007). Integrin alpha11 regulates IGF2 expression in fibroblasts to enhance tumorigenicity of human non-small-cell lung cancer cells. Proc. Natl. Acad. Sci. USA.

[45] Sakiyama T., Kohna T., Mimaki S., Ohta T., Yanagitani N., Sobue T., Kunitoh H., Saito R., Shimizu K., Hirama C. (2005). Association of amino acid substitution polymorphisms in DNA repair genes TP53, POLI, REV1, and LIG4 with lung cancer. Int. J. Cancer.

[46] Zhang X., Miao X., Sun T., Tan W., Qu S., Xiong P., Zhou Y., Lin D. (2005). Functional polymorphisms in cell death pathway genes FAS and FASL contribute to risk of lung cancer. J. Med. Genet..

[47] Zhang Z., Qiu L., Wang M., Tong N., Li J., Zhang Z. (2009). The Fas ligand promoter polymorphisms, rs763110 (-844C<T) contributes to cancer susceptibility: Evidence from 19 case-control studies. Eur. J. Hum. Genet..

[48] Rudd M.F., Webb El Matakidou A., Sellick G.S., Williams R.D., Bridle H., Eisen T., Houlston R.S. (2007). Variants in the GH-IGF axis confer susceptibility to the lung cancer. Genome Res..

[49] Landi M.T., Chatterjee N., Yu K., Goldin L.R., Goldstein A.M., Rotunno M., Mirabello L., Jacobs K., Wheeler W., Yeager M. (2009). A Genome-wide Association Study of Lung Cancer Identifies a Region of Chromosome 5p15 Associated with Risk for Adenocarcinoma. Am. J. Hum. Genet..

[50] Tu H., Heymach J.V., Wen C.-P., Ye Y., Pierzynski J.A., Roth J.A., Wu X. (2016). Different dietary patterns and reduction of lung cancer risk: A large case-control study in the U.S. Sci. Rep..

[51] Wain L.V., Shrine N., Artigas M.S., Erzurumluoglu A.M., Noyvert B., Bossini-Castillo L., Obeidat M.E., Henry A.P., Portelli M.A., Hall R.J. (2017). Genome-wide association analyses for lung function and chronic obstructive pulmonary disease identify new loci and potential druggable targets. Nat. Genet..

[52] DeMeo D.L., Mariani T., Bhattacharya S., Srisuma S., Lange C., Litonjua A., Bueno R., Pillai S.G., Lomas D.A., Sparrow D. (2009). Integration of Genomic and Genetic Approaches Implicates IREB2 as a COPD Susceptibility Gene. Am. J. Human. Genet..

[53] Serveaux-Dancer M., Jabaudon M., Creveaux I., Belville C., Blondonnet R., Gross C., Constantin J.-M., Blanchon L., Sapin V. (2019). Pathological Implications of Receptor for Advanced Glycation End-Product (*AGER*) Gene Polymorphism. Dis. Markers.

[54] Yin N., Lang X., Wang X., Liu W. (2015). AGER genetic polymorphisms increase risks of breast and lung cancers. Genet. Mol. Res..

[55] Zhao D.-C., Lu H.-W., Huang Z.-H. (2015). Association between the receptor for advanced glycation end products gene polymorphisms and cancer risk: A systematic review and meta-analysis. JBUON.

[56] Yamaguchi K., Iwamoto H., Sakamoto S., Horimasu Y., Masuda T., Miyamoto S., Nakashima T., Ohshimo S., Fujitaka K., Hamada H. (2017). AGER rs2070600 polymorphism elevates neutrophil lymphocyte ratio and mortality in metastatic lung adenocarcinoma. Oncotarget.

[57] Oczypok E.A., Perkins T.N., Oury T.D. (2017). All the “RAGE” in lung disease: The receptor for advanced glycation end products (RAGE) is a major mediator of pulmonary inflammatory responses. Paediatr. Respir. Rev..

[58] Huang Q., Mi J., Wang X., Liu F., Wang D., Yan D., Wang B., Zhang S., Tian G. (2016). Genetically lowered concentrations of circulating sRAGE might cause an increased risk of cancer: Meta-analysis using Mendelian randomization. J. Int. Med. Res..

[59] Cheng D.T., Kim D.K., Cockayne D.A., Belousov A., Bitter H., Cho M.H., Duvoix A., Edwards L.D., Lomas D.A., Miller B.E. (2013). Systemic Soluble Receptor for Advanced Glycation Endproducts Is a Biomarker of Emphysema and Associated with AGER Genetic Variants in Patients with Chronic Obstructive Pulmonary Disease. Am. J. Respir. Crit. Care Med..

[60] Trendowski M.R., Lusk C.M., Wenzlaff A.S., Neslund-Dudas C., Gadgeel S.M., Soubani A.O., Schwartz A.G. (2023). Assessing a Polygenic Risk Score for Lung Cancer Susceptibility in Non-Hispanic White and Black Populations. Cancer Epidemiol. Biomark. Prev..

[61] Gorman B.R., Ji S.-G., Francis M., Sendamarai A.K., Shi Y., Devineni P., Saxena U., Partan E., DeVito A.K., Byun J. (2024). Multi-ancestry GWAS meta-analyses of lung cancer reveal susceptibility loci and elucidate smoking-independent genetic risk. Nat. Commun..

[62] Boumtje V., Manikpurage H.D., Li Z., Gaudreault N., Armero V.S., Boudreau D.K., Renaut S., Henry C., Racine C., Eslami A. (2024). Polygenic inheritance and its interplay with smoking history in predicting lung cancer diagnosis: A French-Canadian case-control cohort. EBioMedicine.

