# Supplementary material for: Polygenic Risk Score Is Associated with Developing and Dying from Lung Cancer in the National Lung Screening Trial
Source: J Clin Med. 2025 Apr 30;14(9):3110. doi: 10.3390/jcm14093110 (PMC12073000; doi:10.3390/jcm14093110)
Supplement: Supplementary file 1 [file jcm-14-03110-s001.zip › jcm-3577254-supplementary.pdf]

Supplementary Data

Polygenic risk score is associated with developing and dying from lung cancer in the National Lung Screening Trial.

Robert P Young, MBChB, PhD, DSc[1], Raewyn J Scott, BN, MPH, PhD[1], Callender T, MBChB, MSc[2], Fenghai Duan, PhD[3], Paul Billings MD, PhD[4], Denise Aberle, MD[5], Greg D Gamble, MSc[1].

Supplementary Figure S1. Consort figure of the genetic study subgroup from the NLST.

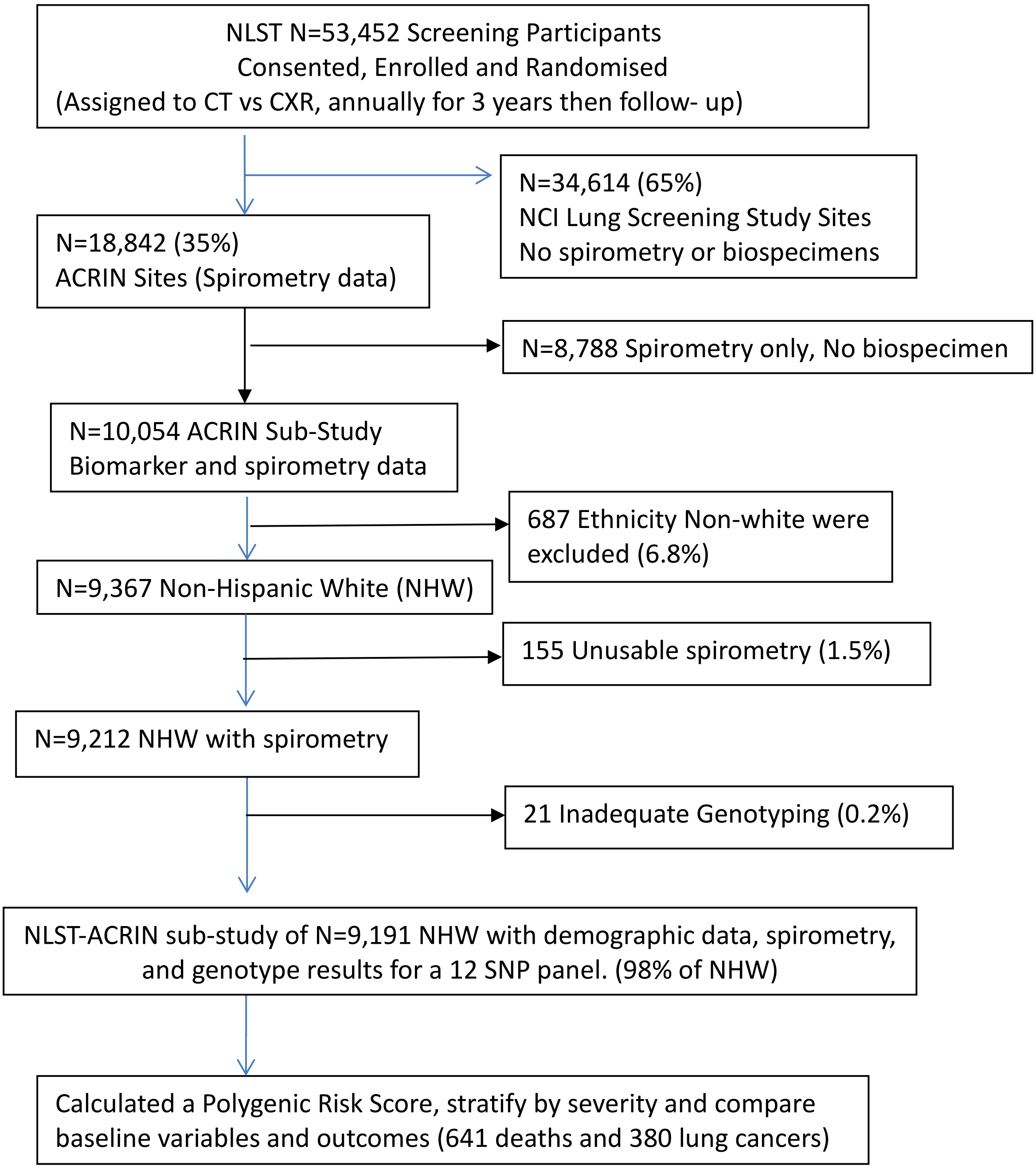

Legend: NLST=National Lung Screening Trial, ACRIN= American College of Radiology, Imaging Network, NCI= National Cancer Institute, NHW=Non-Hispanic Whites, SNP=Single Nucleotide Polymorphism, CT=computerized tomography, CXR=Chest X-ray.

Supplementary Figure S2. Cause-specific mortality analysed across PRS groups as (a) percentage of all deaths and (b) deaths per 1000 within each PRS risk group by crude tertiles.

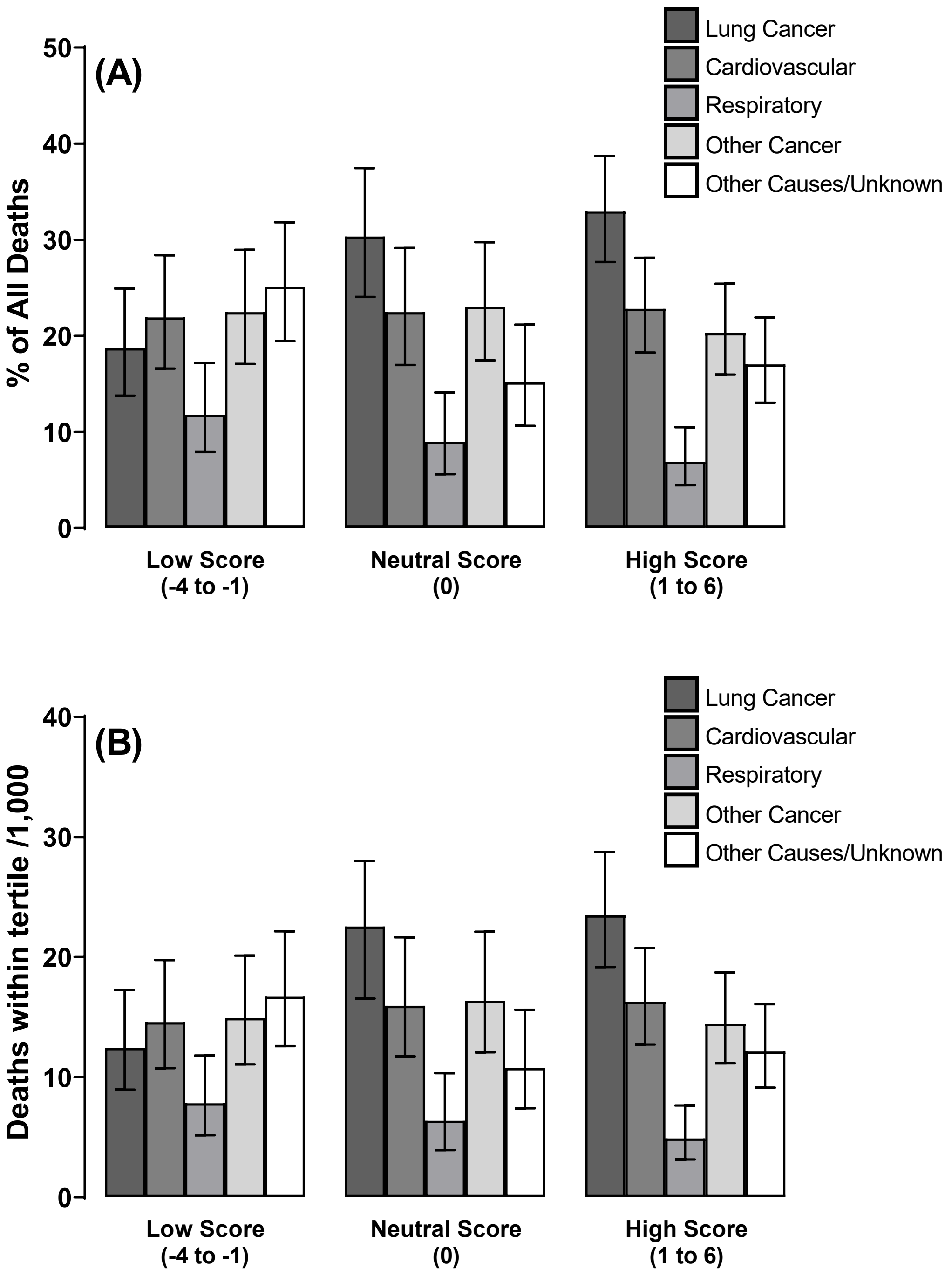

Legend. The relationship between the PRS and cause-specific death suggests an elevated PRS is associated with increasing lung cancer deaths but not total deaths, cardiovascular deaths, respiratory deaths, or other cancer deaths (see also Table 2).

Supplementary Figure S3. Odds of lung cancer deaths referenced against the low PRS risk group and adjusted according to clinical factors, clinical score, and GOLD 1-4 status.

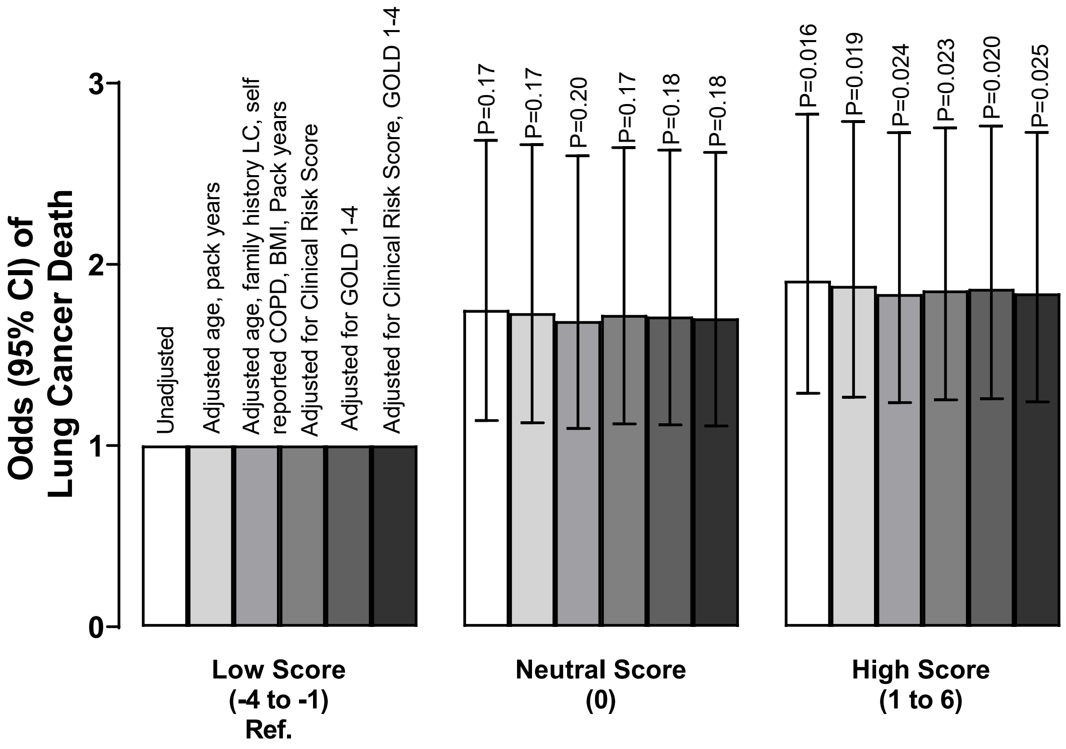

Legend: The odds of lung cancer death has been adjusted for age, pack years, family history of lung cancer, self-reported COPD, BMI, clinical risk score (see Supplementary Table S3) and airflow limitation (COPD GOLD 1-4).

Supplementary Figure S4. Lung cancer prevalence per 1000 using the gene-based (blue), and $PLCO_{M2012}$ (red) models according to quintiles of 6-year risk of developing lung cancer.

(A) Gene-based score and $PLCO_{M2012}$ model in the ACRIN-NLST Sub-study

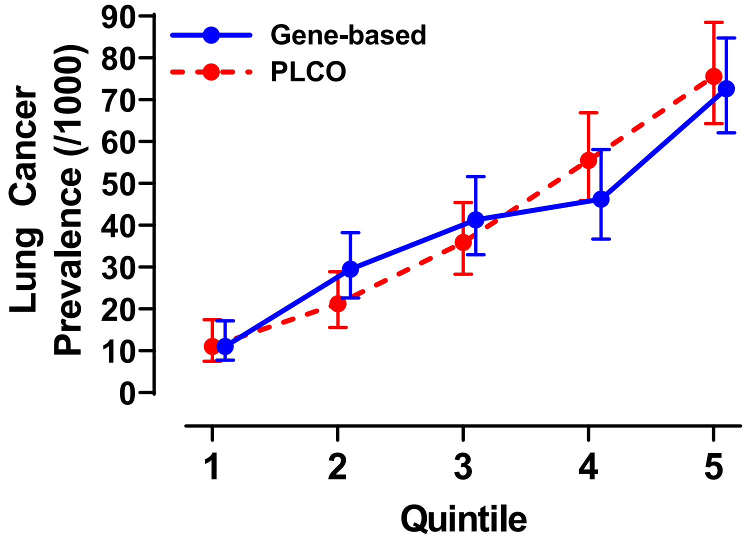

(B) Gene-based score and $PLCO_{M2012}$ model in the UK Biobank (NLST-eligible)

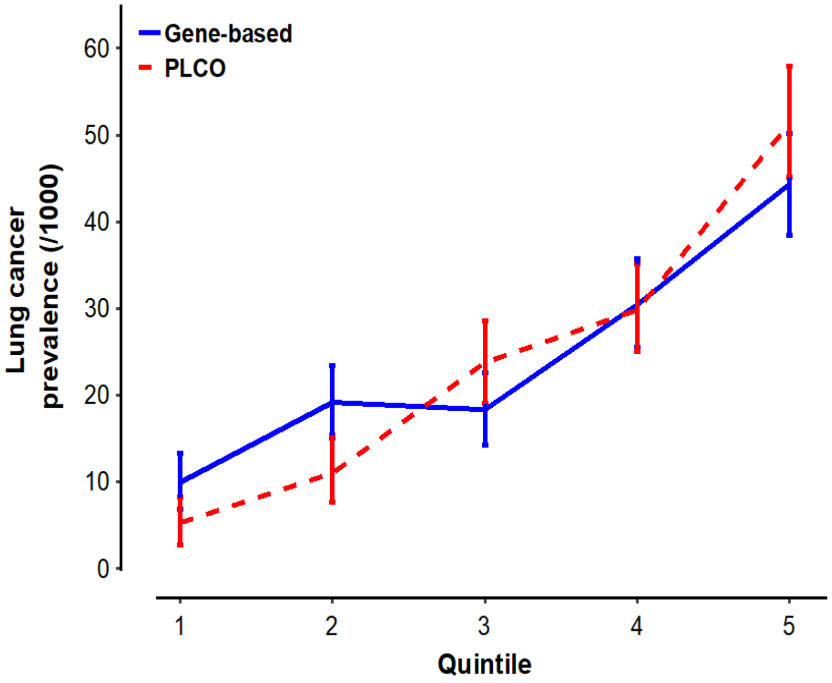

Legend - Gene-based risk score (Supplementary Methods): We have previously described a gene-based risk algorithm that combines clinical variables with our PRS to derive a composite individualised gene-based risk score for developing lung cancer(17,21) (Supplementary Table S2 and References). The predefined scoring algorithm for the clinical variables are age≥65 years old (+4), history of COPD (+4), Family history (1st degree) of lung cancer (+3), abnormal BMI (BMI≤20= +4 and BMI≥25= -1), smoking pack years (pack years ≤39= -2 and pack years ≥60= +2).

Supplementary Table S1. Comparison of the ACRIN sub-cohort (N=9,191), CT arm of the NLST (N=26,723) and NLST-eligible UK Biobank sub-study (N=20,796), according to clinical variables.

**Demographics**	**NLST (CT arm =26,723)**	**ACRIN-NLST (N=9,191)**	**UK Biobank‡ (N=20,796)**
Age (mean ± SD)- yrs	61.4 (5.0)	61.8 (5.1)	62.0 (4.0)
Male Gender %	59%	57%	58%
Current Smoker %	48%	48%	43%
Pack Years (mean ± SD)	56.0 (23.9)	56.2 (23.3)	48.6 (19.7)
Family history of Lung Cancer %	22%	24%	18%
Personal history of COPD %	17%	21%	9%
BMI (mean ± SD)	27.9 (5.0)	27.9 (5.1)	28.4 (5.0)
Education Level<br>- Less than College<br>-College or higher<br>-Other/missing	 43.8%<br>53.9%<br>2.3%	 39.3%<br>58.4%<br>2.3%	 73.3%<br>25.4%<br>1.3%
**Pre-morbid disease (self-report)**			
COPD	1347 (5.0%)	662 (7%)	-
Chronic Bronchitis	2592 (9.7%)	1023 (11%)	-
Emphysema	2056 (7.7%)	847 (9.2%)	-
Asthma -adult	1666 (6.2%)	628 (6.8%)	2462 (11.9%)
Pneumonia	5930 (22.2%)	2580 (28%)	398 (1.9%)
Heart Disease	3445 (12.9%)	1225 (13%)	3076 (14.8%)
Hypertension	9378 (35.1%)	3266 (36%)	8150 (39.3%)
Stroke	753 (2.8%)	266 (2.9%)	953 (4.6%)
Diabetes	2594 (9.7%)	836 (9.1%)	1927 (9.3%)
Any cancer History	1073 (4.0%)	381 (4.2%)	2328 (11.2%)

‡ Meeting NLST-eligible criteria (non-imputed data – see legend below).

Supplementary Table S2. Lung cancer (LC) characteristics according to the lung cancer polygenic risk score (PRS) score (Low, Neutral, High).

Polygenic Risk Score Groups<br>(absolute score range)	**Low Score<br>(-4 to -1)**	**Neutral Score<br>0**	**High Score<br>(1 to 6)**	**P value**
N=9,191 NHW (% of total cohort)	2813 (30.6%)	2504 (27.2%)	3874 (42.2%)	**Total**
Lung Cancers -N‡ (per 1000)	78 (27.7)	102 (40.7)	200 (51.6)	**<0.0001**
Demographics				
Age – mean (SD)	64.2 (5.0)	63.3 (5.2)	64.2 (5.5)	0.37
Sex (% Male)	46 (59.0%)	63 (61.8%)	104 (52.0%)	0.23
Pack Years –mean (SD)	63.6 (29.1)	62.5 (24.0)	64.1 (25.9)	0.88
Smoking Status (%Current)	49 (62.8%)	58 (56.9%)	107 (53.5%)	0.37
**Self-reported Comorbidity**				
- COPD	20 (25.6%)	36 (35.3%)	64 (32.0%)	0.38
-Pneumonia	23 (29.5%)	34 (33.3%)	67 (33.5%)	0.80
-Heart Dx	8 (10.3%)	20 (19.6%)	31 (15.5%)	0.23
-Diabetes	5 (6.4%)	11 (10.8%)	18 (9.0%)	0.59
-Hypertension	33 (42.3%)	37 (36.3%)	74 (37.0%)	0.66
-Stroke	1 (1.3%)	6 (5.9%)	6 (3.0%)	0.38
Lung Function				
FEV_1/FVC -mean (SD)	66.57 (10.76)	66.10 (12.0)	66.39 (11.8)	0.96
FEV_1 % predicted -mean (SD)	71.57 (20.4)	74.99 (20.8)	74.28 (23.0)	0.55
GOLD 1-2	29 (37.2%)	36 (35.3%)	74 (37.0%)	0.95
GOLD 3-4	14 (18.0%)	14 (13.7%)	28 (14.0%)	0.67
Lung Cancer (LC) Characteristics				
Lung Cancer Death N (% of LC)‡	35/78 (44.9%)	54/102 (52.9%)	91/200 (45.5%)	0.43
Lung Cancer Death N (% of total grp)	35/2813 (1.2%)	54/2504 (2.2%)	91/3874 (2.3%)	**0.0040**
Lung Cancer Death during study (% of LC)†	30/73 (41.1%)	49/97 (50.5%)	88/197 (44.7%)	0.45
Lung cancer Histology				
- Small Cell	12 (15.4%)	8 (7.8%)	28 (14.0%)	0.46
- Squamous Cell	18 (23.1%)	26 (25.5%)	42 (21.0%)	
-Adenocarcinoma/BAC$^{\phi}$	29 (37.2%)	46 (45.1%)	90 (45.0%)	
- Non-small /Large Cell	14 (18.0%)	17 (16.7%)	36 (18.0%)	
- Other/unknown	5 (6.4%)	5 (4.9%)	4 (2.0%)	
Lung cancer Stage at Diagnosis N (%)				
- Stage I-II	38 (48.7%)	42 (41.2%)	100 (50.0%)	0.35
- Stage III-IV	34 (43.6%)	53 (52.0%)	93 (46.5%)	
- Occult carcinoma/ unknown	6 (7.7%)	7 (6.9%)	7 (3.5%)	
Surgery - Yes	37 (47.4%)	47 (46.1%)	110 (55.0%)	0.26
Interval lung Cancer diagnosed				
- Screening (T0-T2)	47 (60%)	59 (58%)	123 (61%)	0.83
- Follow up (T3-T7)	31 (40%)	43 (42%)	77 (39%)	
Cancer detection				
- Screen-detected	40 (51.3%)	45 (44.1%)	104 (52.0%)	0.64
- Interval/Missed	7 (9.0%)	14 (13.7%)	19 (9.5%)	
- Follow up	31 (39.7%)	33 (32.3%)	77 (38.5%)	

‡Total lung cancers including those diagnosed at post-mortem (N=13),† Excludes LC diagnosed at post-mortem. $^{\phi}$BAC=Bronchioloalveolar Carcinomas.

Supplementary Table S3. Algorithm and referencing for the polygenic risk score (PRS) and clinical score for lung cancer.

**Clinical score**	**Criteria**	**Score**	**References**
**Age**	≥ 65 yo	+4	17,19-21
**Past history of COPD**	Yes	+4	17,19-21
**Family history of Lung Cancer**	Yes	+3	17,19-21
**Body Mass Index**	≤ 20	+4	17,19-21
	20-24		
	≥ 25	-1	
**Smoking history**	30-39 pack yrs	-2	17,19-21
	40-59 pack yrs		
	≥ 60 pack years	+2	
**Polygenic risk score (PRS) genes**	**Gene (rs #)**	**Score**	
1. Nicotinic Acetylcholine subunit receptor	CHRNA (rs16969968)	AA=+1	8-10,12,17,19,30-32
2 HLA-B-associated transcript 3	BAT3 (rs1052486)	CC=+1	17,19-21,31
3 Telomerase reverse transcriptase	TERT (rs402710)	CC=+1	11,31,33
4 Fas Ligand	FasL (rs763110)	TT=-1	45-47
5 Translesion DNA polymerase	REV1 (rs3087386)	CC=-1	45,48
6 Matrix metalloproteinase-12	MMP12 (rs645419)	GG=-1	51
7 Integrin alpha11	ITGA11 (rs2306022)	TT/TC=-1	43,44
8 Interleukin - 6	IL-6 (rs1800797)	GG=+1	31,32
9 Iron regulatory binding protein	IREB2 (rs2656069)	CC/CT=-1	13,52
10 Cytochrome P450 2A6	CYP2A6 (rs7937)	TT=+1	35,
11 C-reactive protein	CRP (rs 2808630)	CC=-1	31,49
12 Glycosylation end product-specific receptor	AGER (rs2070600)	TT/TC=+1	53-59

Legend: The risk genotypes assigned to each of the 12 SNP variants were predefined and combined in a previously published algorithm to derive a composite polygenic risk score (PRS)[17,19-21]. Weightings for clinical variables were based on previous publications and effect size according to the population distribution. Individuals were scored according to a published algorithm [17,19-21]. In 21 individuals, there were 9 or more missing genotypes, and these individuals were excluded from the analysis (see Figure S1 consort diagram). Of the 12 possible risk genotypes, results were available for all 12 SNPs in 8,629 subjects (94%), 11 SNP genotypes in a further 288 subjects (3%) and 10 or more genotypes in 84 subjects (1%). This means over 98% of all subjects had 10 or more SNP genotypes contributing to their PRS.

Supplementary Table S4. Multivariable logistic regression predicting lung cancer death according to known risk variables including PRS (treated as a continuous variable) in the UK Biobank of ever smokers and those according to USPSTF and NLST screening criteria.

(a) Total UK Biobank ever-smokers (N=167,796)

**Variable**	**Full model**		
	Odds ratio	95% CI	P value
Age	1.09	1.08-1.10	<0.001
Sex (Male vs Female)	0.89	0.83-0.96	0.003
Current Smoker	2.15	1.99-2.31	<0.001
Pack years	1.02	1.02-1.02	<0.001
Family History of LC	1.49	1.34-1.70	<0.001
Self-reported COPD	1.61	1.17-2.21	0.003
BMI	0.98	0.97-0.99	<0.001
High School/Some College	1.47	1.31-1.65	<0.001
12 SNP Lung cancer PRS	1.06	1.02-1.10	0.002
COPD (GOLD 1-4)[#]	1.78	1.56-2.03	<0.001

(b) UK Biobank USPSTF-2021 eligible (N=34,356)

**Variable**	**Full model**		
	Odds ratio	95% CI	P value
Age	1.08	1.07-1.09	<0.001
Sex (Male vs Female)	0.94	0.86-1.03	0.161
Current Smoker	1.60	1.46-1.76	<0.001
Pack years	1.01	1.01-1.01	<0.001
Family History of LC	1.45	1.25-1.68	<0.001
Self-reported COPD	1.33	0.93-1.90	0.121
BMI	0.98	0.97-0.99	0.005
High School/Some College	1.28	1.11-1.49	0.001
12 SNP Lung cancer PRS	1.04	1.00-1.09	0.08
COPD (GOLD 1-4)[#]	1.69	1.44-1.99	<0.001

(c) UK Biobank NLST eligible (N=20,796)

**Variable**	**Full model**		
	Odds ratio	95% CI	P value
Age	1.07	1.05-1.09	<0.001
Sex (Male vs Female)	0.93	0.84-1.04	0.185
Current Smoker	1.49	1.35-1.66	<0.001
Pack years	1.01	1.01-1.01	<0.001
Family History of LC	1.43	1.20-1.69	<0.001
Self-reported COPD	1.35	0.92-1.96	0.122
BMI	0.98	0.96-0.99	<0.001
High School/Some College	1.33	1.12-1.58	<0.001
12 SNP Lung cancer PRS	1.05	0.99-1.10	0.08
COPD (GOLD 1-4)[#]	1.69	1.43-2.00	<0.001

Legend: #COPD (GOLD 1-4) = airflow limitation on pre-bronchodilator spirometry, LC=lung cancer. PRS=polygenic risk score. BMI=body mass index. CXR=chest x-ray, CT=computed tomography.

Supplementary Table S5. Area-under-the curve analyses for 6-year risk of developing or dying of lung cancer in the NLST and UK biobank (validation) sub-groups according to the gene-based composite (PRS + Clinical) score and $PLCO_{M2012}$ model.

**Cohort**	**Outcome (6 yr risk)**	**Variable**	**AUC**	**95% Confidence Interval**
**NLST**				
	Dying of Lung Cancer	PRS alone	0.568	0.528-0.608
		PRS + Clinical Score	0.661	0.623-0.698
	Developing Lung cancer	Clinical alone	0.647	0.620-0.674
		PRS + Clinical score	0.667	0.640-0.693
		$PLCO_{M2012}$	0.677	0.651-0.704
**UK Biobank**	Developing Lung cancer (NLST-eligible, N=20,796)	Clinical alone	0.627	0.607-0.647
		PRS + Clinical	0.628	0.607-0.648
		$PLCO_{M2012}$	0.675	0.654-0.694
	Developing Lung cancer (USPSTF-2021, N=34,356)	Clinical alone	0.643	0.623-0.660
		PRS + Clinical	0.645	0.624-0.662
		$PLCO_{M2012}$	0.705	0.690-0.723

Legend: The discriminatory performance of the composite (PRS +Clinical score) gene-based score is comparable to that of the $PLCO_{M2012}$ in the ACRIN-NLST screening subgroup (overlapping AUC). The discriminatory performance of the composite gene-based score is marginally lower in the NLST-eligible UK Biobank subgroup validation and lower than the $PLCO_{M2012}$ model.
